# Molecular Process Producing Oncogene Fusion in Lung Cancer Cells by Illegitimate Repair of DNA Double-Strand Breaks

**DOI:** 10.3390/biom5042464

**Published:** 2015-09-30

**Authors:** Yoshitaka Seki, Tatsuji Mizukami, Takashi Kohno

**Affiliations:** 1 Division of Genome Biology, National Cancer Center Research Institute, Chuo-ku, Tokyo 104-0045, Japan; E-Mails: yoseki@ncc.go.jp (Y.S.); tmizukam36@gmail.com (T.M.); 2 Division of Respiratory Diseases, Department of Internal Medicine, Jikei University School of Medicine, Minato-ku, Tokyo 105-8471, Japan

**Keywords:** lung cancer, oncogene, fusion, non-homologous end-joining, synthesis-dependent end-joining

## Abstract

Constitutive activation of oncogenes by fusion to partner genes, caused by chromosome translocation and inversion, is a critical genetic event driving lung carcinogenesis. Fusions of the tyrosine kinase genes *ALK* (anaplastic lymphoma kinase), *ROS1* (c-ros oncogene 1), or *RET* (rearranged during transfection) occur in 1%–5% of lung adenocarcinomas (LADCs) and their products constitute therapeutic targets for kinase inhibitory drugs. Interestingly, *ALK*, *RET*, and *ROS1* fusions occur preferentially in LADCs of never- and light-smokers, suggesting that the molecular mechanisms that cause these rearrangements are smoking-independent. In this study, using previously reported next generation LADC genome sequencing data of the breakpoint junction structures of chromosome rearrangements that cause oncogenic fusions in human cancer cells, we employed the structures of breakpoint junctions of *ALK*, *RET*, and *ROS1* fusions in 41 LADC cases as “traces” to deduce the molecular processes of chromosome rearrangements caused by DNA double-strand breaks (DSBs) and illegitimate joining. We found that gene fusion was produced by illegitimate repair of DSBs at unspecified sites in genomic regions of a few kb through DNA synthesis-dependent or -independent end-joining pathways, according to DSB type. This information will assist in the understanding of how oncogene fusions are generated and which etiological factors trigger them.

## 1. Introduction

**Figure 1:**
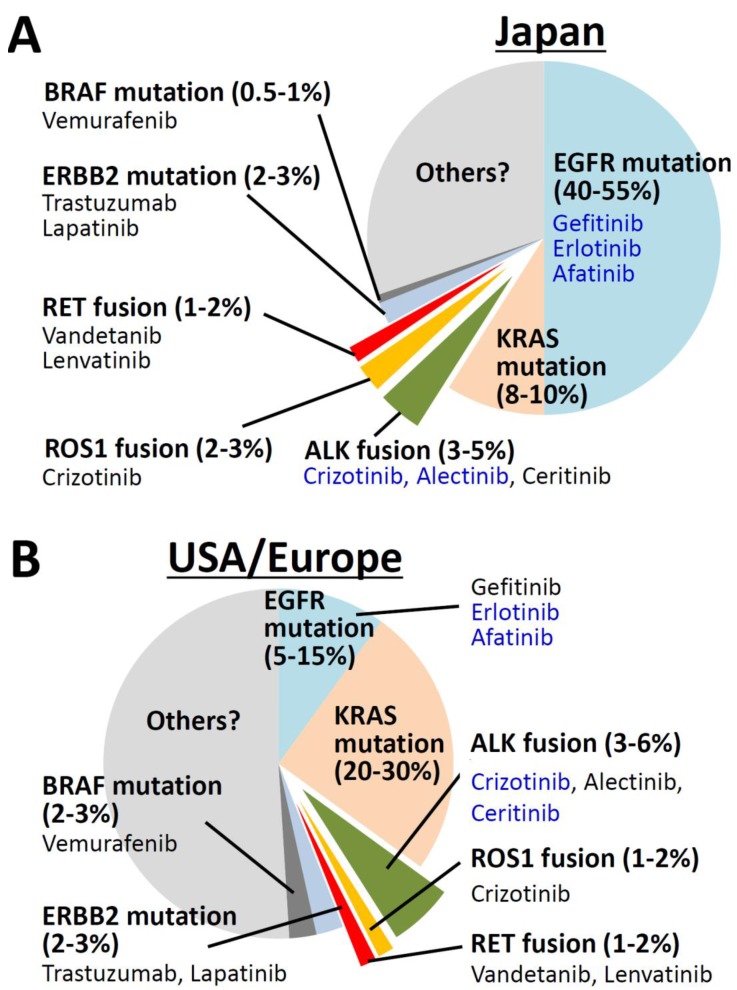
Mutually exclusive occurrence of oncogene aberrations in lung adenocarcinomas (LADC). Data on patients in Japan (**A**) and of European descent (**B**) were generated by summarizing the results of previous reports. Molecular target drugs for each oncogene aberrations are shown in blue (approved for lung cancer) or black (in clinical or preclinical studies). Anaplastic lymphoma kinase (*ALK*), c-ros oncogene 1 (*ROS1*), and rearranged during transfection (*RET*) oncogene fusions are present in a subset of LADCs in both populations.

Fusion of *ALK* (anaplastic lymphoma kinase), *ROS1* (c-ros oncogene 1), and *RET* (rearranged during transfection) oncogenes, which encode tyrosine kinases, with several partner genes by gross chromosome rearrangements is a genetic alteration that drives lung carcinogenesis by causing constitutive activation of these kinases. These gene fusions are mutually exclusive with each other and with mutations of other oncogenes, such as *EGFR* (epidermal growth factor receptor), *KRAS* (Kirsten rat sarcoma viral oncogene homolog), *BRAF* (B-Raf proto-oncogene), and *ERBB2* (*erb-b2 receptor tyrosine kinase 2*), in lung adenocarcinoma (LADC) (Figure 1) [1,2,3]. Although *ALK*, *ROS1*, and *RET* fusions occur in a small subset (1%–5%) of LADCs, they are of particular interest for two reasons. First, drugs that inhibit ALK, ROS1, and RET kinases have marked therapeutic effects on fusion-positive LADCs because the survival and growth of such cancer cells are highly dependent on the kinase activity of fusion proteins. Second, *ALK*, *ROS1*, and *RET* fusions are preferentially detected in never- and light-smokers. Therefore, chromosome rearrangements producing oncogene fusions are likely to be smoking-independent, whereas activating mutations of the *KRAS* oncogene are strongly associated with tobacco smoking [4].

**Figure 2:**
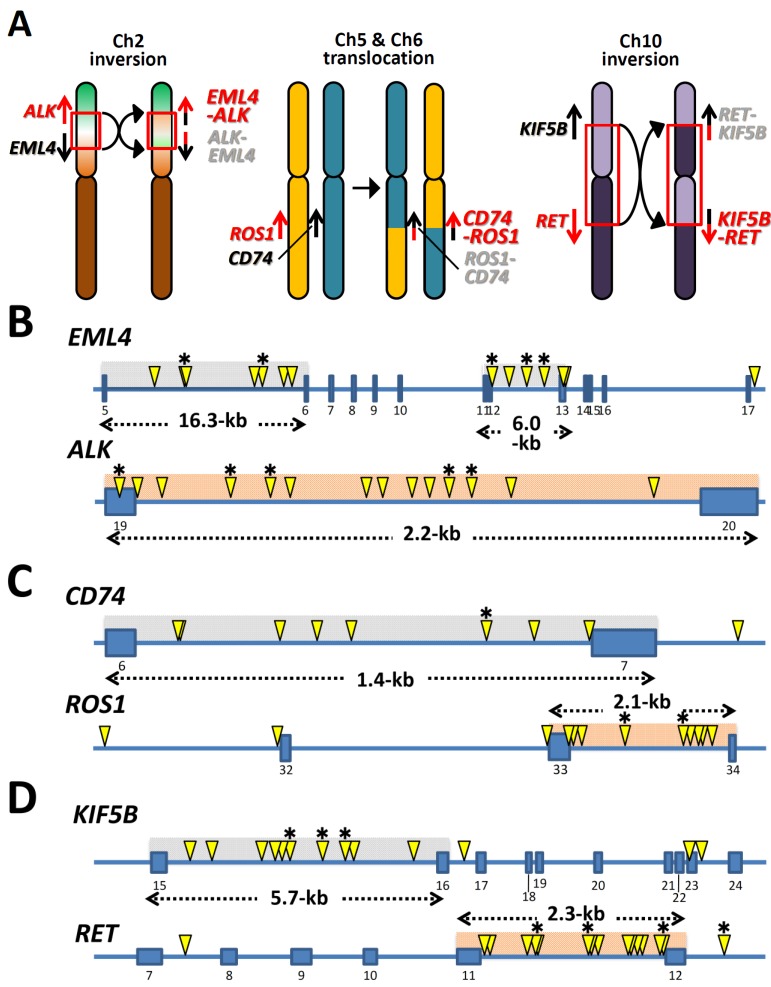
Cluster of breakpoints in oncogenes and partner genes. (**A**) Chromosome inversion and translocation producing oncogene fusions. (**B**–**D**) Distribution of breakpoints in *ALK* and its major partner gene *EML4* (**B**), *ROS1* and its major partner gene *CD74* (**C**), and *RET* and its major partner gene *KIF5B* (**D**). Yellow arrowheads indicate the locations of breakpoints for fusions in 41 Japanese LADC cases. All these cases were identified in a Japanese LADC cohort of 608 cases [8]. Breakpoints for chromosome rearrangements were identified by next-generation sequencing and/or genomic PCR analyses of tumor DNAs as previously described [9]. Breakpoint cluster regions are gray-hatched for partner genes and orange-hatched for oncogenes. Breakpoints in tumors of smokers are marked by asterisks.

LADC is the most frequent histological type of lung cancer in Asian and European countries, and it is less associated with smoking than other types of lung cancers [5]. Therefore, elucidation of the mechanism(s) that causes oncogene fusions may help identify risk factors or preventive methods that could reduce the incidence of LADC. Chromosome rearrangements, such as translocation and inversion (Figure 2A), that produce oncogene fusions are supposedly caused by DNA double-strand breaks (DSBs) and subsequent illegitimate repair (*i.e*., joining) of broken DNA ends. Structural analysis of the breakpoints for such rearrangements is thought to be a powerful way to deduce the molecular processes underlying their occurrence because the breakpoints retain “traces” of DSBs and their subsequent repair [6,7]. This type of analysis provides information about the locations (clustering) of breakpoints on a genomic segment, which enables the identification of the genomic/chromosomal features that make DNA susceptible to DSBs, as well as the structures of breakpoint junctions, which enables the identification of the DSB repair pathways used for illegitimate joining of broken DNA ends. In this review, we summarize the information about breakpoint junctions of *ALK*, *ROS1*, and *RET* fusions obtained in our previous genomic analyses [3,8,9,10,11,12], and examined how oncogene fusions are generated in the course of carcinogenesis.

## 2. Distribution of Breakpoints in Oncogenes and Partner Genes

The location (clustering) of the breakpoints for chromosome rearrangements of *ALK*, *ROS1*, and *RET* fusions is illustrated in Figure 2B–D (detailed data in Supplementary Tables 1–2 and Supplementary Figure 1). As we reported for the *RET* fusion [9], breakpoints in the *ALK* and *ROS1* oncogenes are also clustered within a defined region of a few kilobases (kb) in size. Breakpoints in partner genes were also mapped within a defined region of several kb in size. Interestingly, none of the breakpoints were mapped at the same position. The location of breakpoints does not necessarily coincide with the location of DNA breaks because broken DNA ends are often enzymatically resected before joining [13,14]. However, the high diversity in the location of breakpoints indicates that DSBs triggering oncogenic *ALK*, *ROS1*, and *RET* fusions in LADC preferentially occur in a few defined regions, but at non-specific sites within these regions. The breakpoint locations were not apparently affected by the smoking history of patients; therefore, DNA damage due to smoking is unlikely to be an important factor for DSB formation (Figure 1). The breakpoint cluster regions lack repetitive sequence clusters and have an average GC content. Furthermore, histone modifications in these regions in cultured non-cancerous lung epithelial cells (SAEC: Human Small Airway Epithelial Cells) indicate they have a closed chromatin structure (http://dbtss.hgc.jp).

## 3. Canonical Non-Homologous End-Joining (NHEJ), a Major DSB Repair Pathway for Illegitimate Joining of DNA Ends

The structures of breakpoint junctions were studied to deduce the DNA repair pathways involved in the joining of broken DNA ends. To precisely deduce the molecular process of joining, including DNA end resection and duplication, reciprocal gene fusion cases were chosen for this analysis, such as a case in which both oncogenic *EML4-ALK* and non-oncogenic *ALK-EML4* fusion DNA was retained in tumor cells (Table 1). Consistent with the findings to date [15,16], only about one-third (15/41, 37%) of cases had reciprocal fusions, while the remaining 26 cases retained only oncogenic fusion DNAs. In the reciprocal cases, nucleotide deletions occurred frequently (11/15; 73%) in the oncogene and/or partner gene loci during the joining (Table 1).

**Figure 3:**
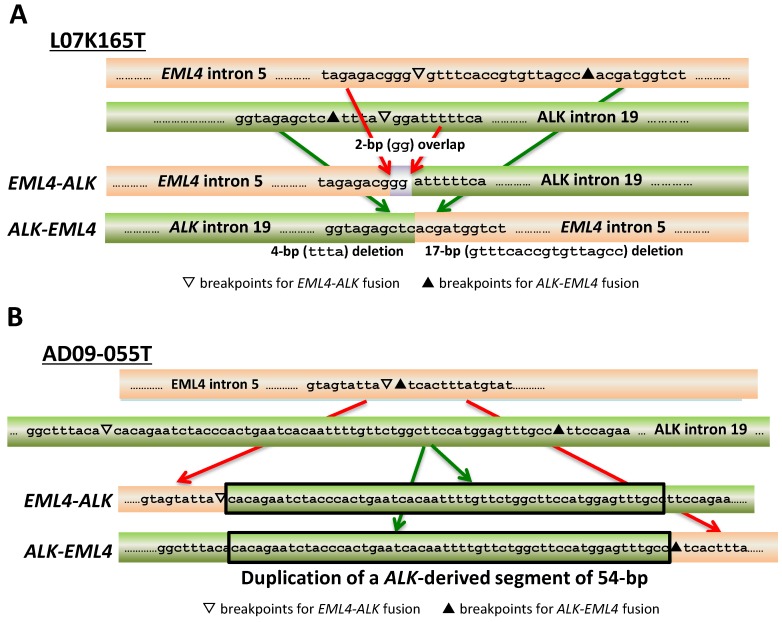

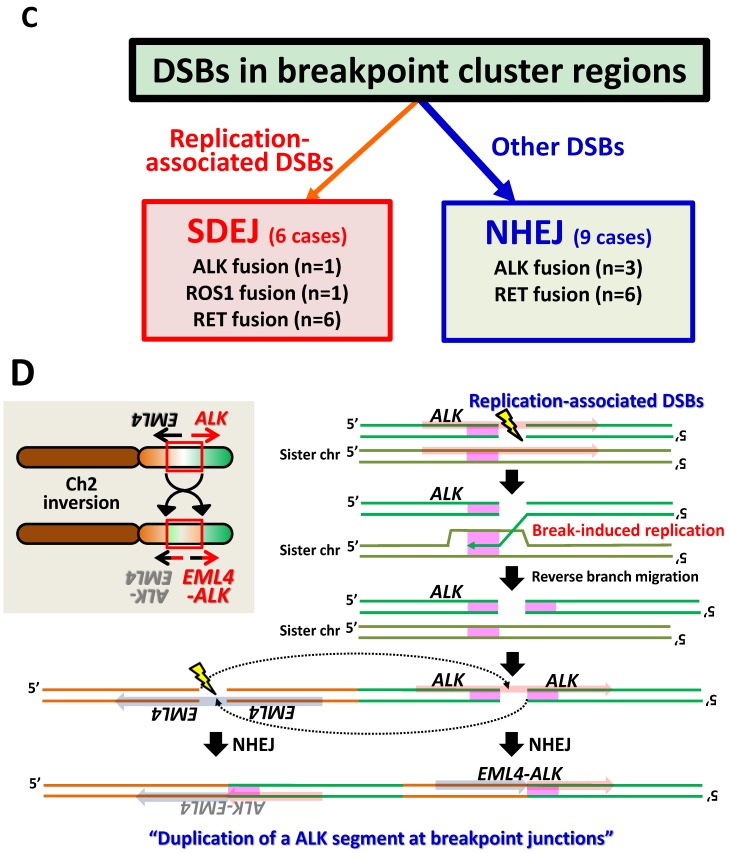
Deduction of joining repair pathways based on the structure of breakpoint junctions. (**A**) A representative case in which reciprocal fusion was determined to be caused by non-homologous end joining (NHEJ). Overlapping and deleted nucleotides at breakpoint junctions are indicated; (**B**) A representative case in which reciprocal fusion occurred with duplication of a genomic segment (indicated by black rectangle); (**C**) Molecular processes causing gene fusions in LADC. Illegitimate DSB repair by synthesis-dependent end-joining (SDEJ) and NHEJ produces oncogene fusion. The pathway used might depend on the type of DSB, *i.e.*, replication-associated or not; (**D**) Deduced process of reciprocal *EML4-ALK* fusion by SDEJ causing duplication of an *ALK* genome segment at breakpoint junctions, as in the case of AD09-055T (Figure 3B).

**Figure 4:**
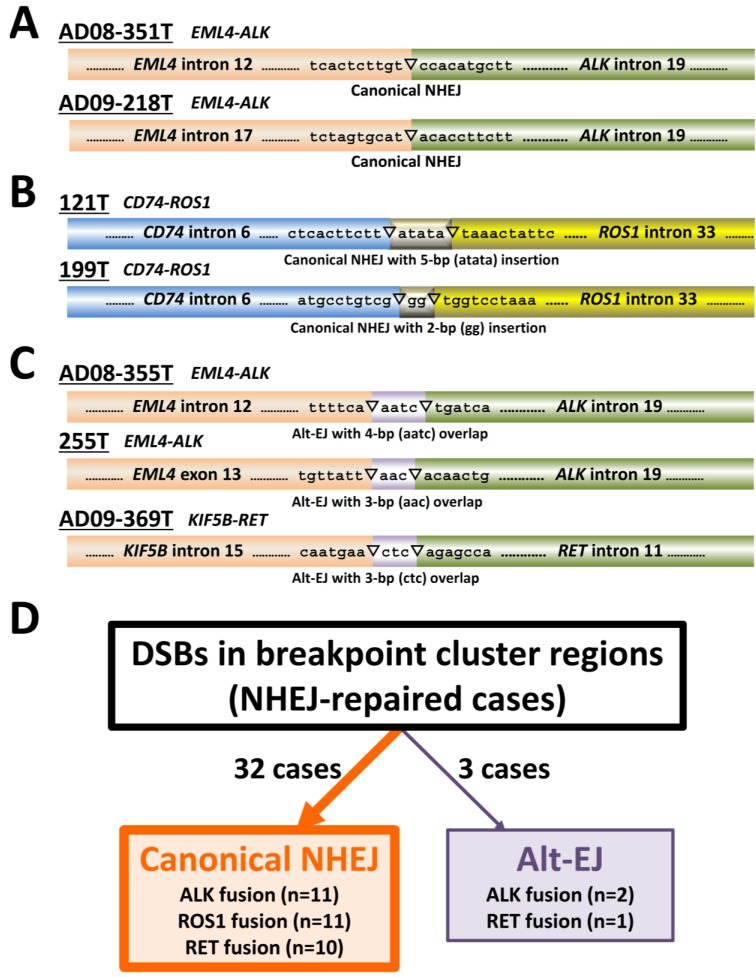
Deduction of non-homologous end joining (NHEJ) modes based on the structures of breakpoint junctions. (A and B) Representative cases in which reciprocal fusion was deduced to be caused by canonical NHEJ. Joining was performed without (**A**) and with (**B**) nucleotide insertions. (**C**) Representative cases in which reciprocal fusion was deduced to be caused by alt-EJ. Joining was deduced to involve microhomologies of three or four base pairs. C, NHEJ causing gene fusions in LADC. Canonical NHEJ is a major NHEJ mode used for DNA end-joining.

The structures of the breakpoint junctions indicated the involvement of two DSB repair pathways in the illegitimate joining of broken DNA ends. One is NHEJ, which joins DNAs that have extremely low (a few bp) or no homology between DNA ends and often inserts a few nucleotides at the junctions [13,14]. Nine (60%) of the 15 cases showed this feature (a representative case is shown in Figure 3A). NHEJ has canonical and non-canonical forms; in the latter, called alternative end-joining (alt-EJ), DNA ends are joined using microhomology of a few nucleotides, leaving an overlap of a few nucleotides at breakpoint junctions [13]. In these nine cases, joining was judged to be achieved by canonical NHEJ because overlap of nucleotides of three or more bp was not detected (Table 2). In the 26 non-reciprocal cases, the detailed joining mechanisms could not be deduced due to a lack of sequence information from breakpoints in reciprocal counterparts; however, overlap of nucleotides of three or more bp was detected in only three cases (12%) (Table 2, representative cases are shown in Figure 4A–C). In total, 32/35 cases (91%) showed NHEJ involvement without the need for DNA end microhomologies (Figure 4D). Thus, canonical NHEJ, but not alt-EJ, is a major DNA repair pathway for illegitimate DNA end-joining producing gene fusions. This is consistent with a recent study of chromosome translocations triggered by artificial DSBs, which reported that canonical NHEJ, but not alt-EJ, is responsible for chromosome translocations in human cells, although the opposite is true in murine cells [17]. Interestingly, translocation junctions in blood tumors, which are driven by oncogene fusion, often lack microhomology at breakpoint junctions [18,19,20,21]. Thus, canonical NHEJ is likely to be a common DNA repair pathway for the illegitimate DNA end-joining that produces gene fusions in a variety of tumors.

**Table 1:** Structure of breakpoint junctions for *ALK*, *ROS1*, *and RET* reciprocal fusions in lung adenocarcinoma.

Onco-gene	Sample name	Partner gene	Nucleotide deletion		DNA segment duplication		Mode of DNA end joining	Smoking
			Kinase	Partner	Kinase	Partner		
ALK	L07K165_T	EML4	4-bp	17-bp	-	-	NHEJ	No
	AD09-357T	EML4	4-bp	-	-	-	NHEJ	No
	L07K154_T	EML4	-	-	-	-	NHEJ	No
	AD09-055T	EML4	-	-	54-bp	-	SDEJ	No
ROS1	103T	CD74	-	32-bp	41-bp	-	SDEJ	No
RET	BR0020	KIF5B	-	-	-	-	NHEJ	No
	L07K201T	KIF5B	15-bp	19-bp	-	-	NHEJ	Yes
	349T	KIF5B	1-bp	7-bp	-	-	NHEJ	Yes
	AD08-341T	KIF5B	16-bp	26-bp	-	-	NHEJ	No
	RET-024	CCDC6	14-bp	2-bp	-	-	NHEJ	Yes
	RET-030	CCDC6	52-bp	1021-bp	-	-	NHEJ	No
	AD12-106T	KIF5B	-	573-bp	490-bp	-	SDEJ	Yes
	BR0030	KIF5B	-	-	-	211-bp	SDEJ	No
	442T	KIF5B	269-bp	-	-	235-bp	SDEJ	No
	AD08-144T	KIF5B	7-bp	-	-	2576-bp	SDEJ	No

**Table 2:** Structure of breakpoint junctions for *ALK*, *ROS1*, and *RET* fusions determined to be caused by NHEJ.

Onco-gene	Sample name	Partner gene	Reciprocal	Nucleotide overlap at junction	Nucleotide insertion at junction	Mode of NHEJ	Smoking
ALK	L07K165_T	EML4	yes	GG	-	C-NHEJ	No
	AD09-357T	EML4	yes	T	-	C-NHEJ	No
	L07K154_T	EML4	yes	-	AC	C-NHEJ	No
	43T	EML4	no	T	-	C-NHEJ	Yes
	137T	EML4	no	CT	-	C-NHEJ	Yes
	169T	EML4	no	-	-	C-NHEJ	Yes
	236T	EML4	no	TA	-	C-NHEJ	No
	255T	EML4	no	AAC	-	Alt-EJ	No
	L07K098_T	EML4	no	A	-	C-NHEJ	No
	AD08_351T	EML4	no	-	-	C-NHEJ	Yes
	AD08_355T	EML4	no	AATC	-	Alt-EJ	No
	AD09-218T	EML4	no	-	-	C-NHEJ	No
	AD09-352T	EML4	no	-	-	C-NHEJ	Yes
ROS1	121T	CD74	no	-	ATATA	C-NHEJ	No
	199T	CD74	no	-	GG	C-NHEJ	No
	L07K147_T	EZR	no	-	-	C-NHEJ	No
	AD08_009T	CD74	no	-	-	C-NHEJ	No
	AD08_034T	EZR	no	T	-	C-NHEJ	No
	AD08_047T	CD74	no	CA	-	C-NHEJ	No
	AD09-074T	CD74	no	-	-	C-NHEJ	No
	AD09-224T	CD74	no	-	-	C-NHEJ	No
	AD09-230T	CD74	no	AT	-	C-NHEJ	No
	AD09-254T	CD74	no	AC	-	C-NHEJ	Yes
	AD09-466T	EZR	no	-	T	C-NHEJ	Yes
RET	BR0020	KIF5B	yes	-	-	C-NHEJ	No
	L07K201T	KIF5B	yes	C	ATA	C-NHEJ	Yes
	349T	KIF5B	yes	-	A	C-NHEJ	Yes
	AD08-341T	KIF5B	yes	-	-	C-NHEJ	No
	RET-024	CCDC6	yes	-	-	C-NHEJ	Yes
	RET-030	CCDC6	yes	-	-	C-NHEJ	No
	BR1001	KIF5B	no	-	AGT	C-NHEJ	No
	BR1002	KIF5B	no	A	-	C-NHEJ	No
	BR1003	KIF5B	no	-	CTTT	C-NHEJ	No
	AD09-369T	KIF5B	no	CTC	-	Alt-EJ	No
	AD12-001T	KIF5B	no	-	-	C-NHEJ	Yes

## 4. Synthesis-Dependent End-Joining (SDEJ), another DSB Repair Pathway for Illegitimate Joining of DNA Ends

The structures of breakpoint junctions in the other six (40%) reciprocal cases indicated that another DSB repair pathway is responsible for illegitimate joining of broken DNA ends (Table 1). In these cases, DNA segments of 33–490 bp from either the oncogene or partner gene locus were retained at both the partner-oncogene and oncogene-partner fusion breakpoints, resulting in duplication of these segments (Figure 3B). The duplication was observed in all the *ALK*, *ROS1*, and *RET* fusions, indicating a significant contribution of this repair pathway to chromosome rearrangements producing gene fusions. In fact, such duplication at breakpoint junctions was also observed in translocations in an experimental model using human cells [17].

The most likely pathway for joining that causes segmental duplication is SDEJ (Figure 3C), in which a broken DNA end, produced by replication-associated DSBs, initiates synthesis on the sister chromatid after strand invasion in a process called break-induced replication (BIR) [22,23]. Reversed branch migration of the Holliday junction formed following strand invasion can release the invaded strand, which contains extra DNA material from the sister chromatid and is fused to the broken DNA of a different chromosome locus by NHEJ (Figure 3D). Involvement of such a repair pathway has also been suggested to be involved in the formation of *BRAF* fusions in a few pediatric brain tumors based on the finding that the breakpoint junction retains duplicated segments [24]. Thus, SDEJ might be a common mechanism for chromosome rearrangements producing gene fusions.

The mode of joining was not apparently affected by the smoking history of patients; therefore, DNA damage due to smoking is unlikely to be an important factor for repair pathway selection (Table 1). However, SDEJ is triggered by replication-associated DSBs, while NHEJ repairs any kind of DSB. Therefore, it can be speculated that replication-associated DSBs cause gene fusion by SDEJ, while other DSBs, including those in non-replicating cells, cause gene fusion by canonical NHEJ (Figure 3C).

## 5. Molecular Process for Chromosome Rearrangements Producing Gene Fusion

The structure of breakpoint junctions for *ALK*, *ROS1*, and *RET* fusions in LADCs enabled us to deduce the molecular process underlying the chromosome rearrangements that produce gene fusions. First, DSBs occur in a few defined regions, but at non-specific sites within these regions. DSBs are generated both in replicating and non-replicating cells. Second, illegitimate repair of DNA ends by canonical NHEJ or SDEJ causes chromosome rearrangements that produce gene fusion, depending on the type of DSBs (Figure 3C).

The environmental and endogenous factors inducing DSBs that trigger rearrangements remain unknown. However, the contribution of both NHEJ and SDEJ to end joining indicates that a variety of DSBs, including those produced by replication stress, increase the risk of gene fusion. Interestingly, a recent study suggested that the breakpoint cluster regions in *RET* are easily broken during replication through that actions of DNA topoisomerase [25]. In addition, immunohistochemical studies of lung tumor specimens indicate that large amounts of DSBs are produced in pre-malignant lung epithelial cells through replication stress; these cells are thought to be negative for oncogene aberrations, and the DSBs are considered to cause genome instability [26]. *ALK*, *ROS1*, and *RET* fusions are believed to be the “first hit” oncogene aberrations driving lung carcinogenesis [2,8]; therefore, such DSBs might trigger the illegitimate DSB repair that results in chromosome rearrangements and cause malignant transformation of pre-malignant cells.

## 6. Conclusions

Cancer cells carry many different types of genetic aberrations, including mutations and gross chromosomal rearrangements, the latter of which include chromosomal deletions, insertions, inversions, and translocations. A small subset of these aberrations function as “drivers” of carcinogenesis, whereas the remaining variations are “passengers” that accumulate as a consequence of cancer cell genome instability. Recent genome-wide sequencing studies, such as the analysis of 140 cases of non-lymphoid malignancies, including 19 lung cancer cases, enabled the identification of many of the DNA repair pathways that contribute to the formation of gross chromosomal rearrangements as a whole [6]. However, only limited mechanistic information is available about the rearrangements that function as drivers for the development of solid tumors. Interestingly, some oncogenic fusions, such as *ETS* fusions in prostate cancer, are caused by closed chain events involving rearrangements of “non-oncogenic loci” [27]. Here, we provide information about the molecular processes that drive oncogenic fusions, based on a study of *ALK*, *ROS1* and *RET* fusions in LADC. A comparative study of the present information with that obtained previously from other driver rearrangements, as well as from rearrangements at non-oncogenic loci, should help determine more precisely how triggering of DNA damage causes cancer cells to develop and identity the factors that cause driver gene aberrations.

## Supplementary Files

Supplementary File 1
