# Supplementary material for: Molecular Process Producing Oncogene Fusion in Lung Cancer Cells by Illegitimate Repair of DNA Double-Strand Breaks
Source: Biomolecules. 2015 Sep 30;5(4):2464–76. doi: 10.3390/biom5042464 (PMC4693243; doi:10.3390/biom5042464)
Supplement: Supplementary File 1 [file biomolecules-05-02464-s001.pdf]

Supplementary Materials

L07K165T

EML4-ALK tttttatttttttattttttcatatttgtagtagagacg[gg]atttttcaaaaccattttatgttggtgaattcatttc
2-bp (gg) overlap
ALK-EML4 gtaggagtgataatggtcactcacattggtagagctc|acgatggtctcaatctcctgacctcatgatctgcccat

AD09-357T

EML4-ALK ttaggattatagctgtgagccgccgcacctggcctacatt[t]cgttatgctagtccctggaattgggtggggtggtgatt
1-bp (t) overlap
ALK-EML4 gtgtctcaggctgtgccacaggtgccaaggtgtcactt|gagatggaaaataagtgtcacatgatctcattatttct

AD09-055T

EML4-ALK agtaggtagtatta|<**cacagaatctacccactgaatcacaattttgttctggcttccatggagtttgcc**>ttccagaacatcc
Duplication of a *ALK*-derived segment of 54-bp
ALK-EML4 ggctgggctttaca<**cacagaatctacccactgaatcacaattttgttctggcttccatggagtttgcct**>|cactttatgtat

L07K154T

EML4-ALK taacctgtataaccataacttggatccctacatctgggct|actcatttgccctggcccatgtgttgggggctgggc
ALK-EML4 attgcatagaggagggaactgagcacatagcgggtg[ac]ttgtccaagtattctctgtatttttatttattttattt
2-bp (ac) overlap

43T

EML4-ALK acacttaaaagagtaaactatacaaataaggacaaggaaa[t]tcagcctgtgagccaaggtgagctgacctgcaggtcac
1-bp (t) overlap

137T

EML4-ALK ccactgtgcctggctagttctaaaatttacatactgtcc[ct]CTGCCCTCGTGGCCGCCCTGGTCCTGGCTTTCTCCG
2-bp (ct) overlap

169T

EML4-ALK actcatttctggtaattctcacatagtactctttcagtcc|ag**c**ggttagggaaacagggcaggagttaccatccctgcct

236T

EML4-ALK AATTCTGTGGGATCATGATCTGAATCCTGAAAGAGAAA[TA]ctgctggcagagacatgcccaggacagatgggcagag
2-bp (TA) overlap

255T

EML4-ALK TGTTCACACTTTGTCAGATGAGAAATGGGATGTTATT[AAC]acaactgaccaagatcccagctgcaccctcaaatcca
3-bp (AAC) overlap

L07K098T

EML4-ALK caagagcgaaactctcttggaaaaaaaaaaaatgtattg[a]gagccccagggactcccaagggggcaggaaggcagga
1-bp (a) overlap

AD08-351T

EML4-ALK tttgtttgtttgtttttttgagatgg**g**gtttcactcttgt|ccacatgcttccaccggcgcagctcctgtttggctccc

AD08-355T

EML4-ALK tctgtacgaaattaatgttttaattaaatgtttttca[aatc]tgatcacggtcggtccattgcatagaggagggaact
4-bp (aatc) overlap

AD09-218T

EML4-ALK attgtataggcccttcaagtcctttagaatctagtgcat|acaccttctttaatcattttattagtatttctaagtat

AD09-352T

EML4-ALK tgttagagatgagaagaaagggtgaggtttgtgattaag|cctggtcctcatggctcagcttgtaagtaacaagaggc

Figure S1. *Cont.*

103T

CD74-ROS1 ccccttccctctaggattca|<**agcaaaaacaccttgcttttgatttcacatggcataaacac**>tgtctgtatggatgctttca

Duplication of a *ROS1*-derived segment of 41-bp

ROS1-CD74 tgttgctctctgcaaaaaaa<**agcaaaaacaccttgcttttgatttcacatggcataaacac**>|tgcctggggcctcagtttct

121T

CD74-ROS1 agcaccgcggaatctcccatcctctcagctctcacttctt|atata|taaactattccattcatgtgatgtgatctttctaaaacat

5-bp (atata) insertion

ROS1-CD74 cagagctcactcactcactgtgtcctcatcatgcctgtcg|gg|tggtcctaaatttattaaaaagatatatatgtttcctaag

2-bp (gg) insertion

L07K147_T

EZR-ROS1 tgtgtctttgcagAGCTCTCGGAGCAGATTCAGAGGGCCC|cccacgtgtttccattttccccatctcctcctggtactcc

AD08-009T

CD74-ROS1 gcactcatatttgttcacttatccgtcagtcctttcatct|tctacacaactgaaactacctaagagaaattaccatgttt

AD08_034T

EZR-ROS1 accaggaaggaaaccaagaacgtgtcccacagcgcagac[t]atcagtacattcttaacattagcaatagataatggtg

1-bp (t) overlap

AD08_047T

CD74-ROS1 caggctggtcttgaactcctgacctcaagtgatccgcc[ca]tatcaaccaatatttctttttttccctaaagCTGGA

2-bp (ca) overlap

AD09-074T

CD74-ROS1 ctgccaagagagccttgggcgtttcccacctcatggaca|TGGAGgtatgttaccatgtctgtctacacactagctta

AD09-224T

CD74-ROS1 cctggtcccagcaccgcggaatctcccatcctctcagctctc|aatgccaactatttagtatccaaagactgagatttcttgg

AD09-230T

CD74-ROS1 tcagtttctgcatcagattcatagaaggcaccttacatgct[at]tgtcatgtatattttctgtatttattatttttttgcagAA

2-bp (at) overlap

AD09-254T

CD74-ROS1 aattggacacatggccattcacttccttttttgatctcag[ac]aaaatcaggctatagccagatttggtcctcatgccatagt

2-bp (ac) overlap

AD09_466T

EZR-ROS1 agaacgtgtcccacagcgcagactcacagccgaaatgataaa|t|aatttagttgaagcacaggctggata

1-bp (t) insertion

BR0020

KIF5B-RET tggagtttcaccgtgttagccaggatggtcttgatctcca|cctgggctgggcctggtgctcatttagtcctggggcaggggtc
RET-KIF5B ggctatgtagagaagttgtcctggacacttccactgtagt|tgacctctttatctgcccacctcggcctcccagagtgctgggatt

L07K201T

KIF5B-RET actatgttagctttgtaaggaactcccaaatggtctt[c]atagGAGGATCCAAAGTGGGAATTCCCTCGGAAGAACTT

1bp (c) overlap

RET-KIF5B cctcccctgtcatcctcacactttccccccctcttctccccc|ata|tgcattcctatcagccatgaatgagagtctgctgttccac

3-bp (ata) insertion

349T

KIF5B-RET tgtccaggctggtttcgaactcctgactacaggtggtccgcct|a|ggtgtgagtgaacggtgagccacgcagcttatggtggcgt

1-bp (a) insertion

RET-KIF5B aagccaagcccagttctggaagtaacagaggctcagagccaa|a|cctcccaaagtgctggtactacaggcatgagccactg

1-bp (a) insertion

Figure S1. *Cont.*

AD08-341T

KIF5B-RET ggctcactgcaagctcctcttcctgggttcaagcaattctt|cctctcctggtggtggcctgccccttcagtgttcctactagcact
RET-KIF5B cctccaggctgtgccgagtatcctggagcctccttgtcccgg|actacaggctcccactaccacacctggctaatttttg

RET-024

CCDC6-RET gggggaataaacaggactaagacagagactgagatg|tatttgagaggatcaggttgatgtcgccctcatgtcoc
RET-CCDC6 tcagctccagagtcacactcatcagcaccaggtcttggaccca|actgcgtgtaatatggggagaaatctgcagggagaagtca

RET 0030

CCDC6-RET atgtatcataatttatctcttcacctcctgtagggcatct|agttgtcctggacacttccactgtagtcagaggtcctggg
RET-CCDC6 gagggccagtggcagcccttgaggagcagtgcttccacac|gtagcttttgtatgttaggccagatctgttggtttgtcct

AD12 106T

KIF5B-RET ttcttttcctctcatcatatataagctctgttagag|<**aggatcaggtt......470-bp...gagaagttgt**>cctggacacttccactg
Duplication of a *RET*-derived segment of 490-bp
RET-KIF5B caggtcttggacccatgactcaacctcagtatttgag<**aggatcaggtt......470-bp......agaagttgt**>|catacagtatgtatat

BR0030

KIF5B-RET ttttttcctagt<**ctagctgcag......191bp......tcacaaggag**>|gggtgcgtgagggccagtggcagcccttgaggagcagtgcttcc
Duplication of a *KIF5B*-derived segment of 211-bp
RET-KIF5B tgggagggcagggtggtagtgccagcaggcagggtgagc|<**ctagctgcag......191bp......tcacaaggag**>ggaagatggtaacgtt

442T

KIF5B-RET AGAACTTCAGACTTTACACA<**ACCTGCGCAAACT......212bp......ATGACACCGG**>|tccctgcggggcagggaagatcccctgccctcc
Duplication of a *KIF5B*-derived segment of 235-bp
RET-KIF5B ggtgccgagcctctggcggtgccaagcctcacaccaccccc|<**ACCTGCGCAAACT......212-bp......ATGACACCGG**>AGGCAGCGCT

AD08-144T

KIF5B-RET ctgcgcccggcctg<**ttgttgttct ...2556-bp...... acagttttat**>|gagagggccagtggcagcccttgaggagcagtgcttcca
Duplication of a *KIF5B*-derived segment of 2576-bp
RET-KIF5B ggtgggagggcagggtggtagtgccagcaggcagggtgagcg|<**ttgttgttct ...2556-bp......**
acagttttat>ttcttcctgaaggt

BR1001

KIF5B-RET attagttcacggaccagacatagtggctcaagcctgag|tga|agccatggcagtgtcgacactgaccttgactgtgggttccc
3-bp (tga) insertion

BR1002

KIF5B-RET gtttacagttttatgttacagtttacaatgattgttgt[a]ggccagctggggagacagaggccatcctgtgaggggctgcca
1-bp (a) overlap

BR1003

KIF5B-RET tttatcagaaattatataaagaaaccataagtaact|cttt|tttccaacatagGAGGATCCAAAGTGGGAATTCCCTCGGA
4-bp (cttt) insertion

AD09 369T

KIF5B-RET aatttcaaacatataatagtagagagaataatacaatgaa[ctc]agagccaagggtgtgagtgaacggtgagccacgcagctt
3-bp (ctc) overlap

AD12 001T

KIF5B-RET gaacttctggcaaccactgatgtttttactatctttatagtat|gagaccaccaccctaaccccagtcagctccagagtcacact

Figure S1. Structures of breakpoint junctions for *ALK*, *ROS1* and *RET* fusions in lung adenocarcinoma. Nucleotide sequences of partner and kinase genes are indicated in blue and red. Exonic and intronic sequences are indicated by capital and non-capital letters. Locations of breakpoints are indicated by "|". Inserted and overlapped nucleotides at breakpoint junctions are indicated by [nnn] and |nnn|, respectively. Duplicated segments at breakpoints are indicated by <nnn>.

Table S1. Characteristics of lung adenocarcinomas with RET fusion subjected to breakpoint analysis.

Onco-gene	Name	Sex	Age	Smoking (Brinkman index)	Fusion	DNA source	Method for breakpoint identification
ALK	L07K165_T	Female	66	Never	EML4-ALK	Frozen tissue (surgical specimen)	NGS
	AD09-357T	Female	58	Never	EML4-ALK	Frozen tissue (surgical specimen)	NGS
	L07K154_T	Female	55	Never	EML4-ALK	Frozen tissue (surgical specimen)	NGS
	AD09-055T	Female	49	Never	EML4-ALK	Frozen tissue (surgical specimen)	NGS
	43T	Male	61	Ever-smoker (15)	EML4-ALK	Frozen tissue (surgical specimen)	NGS
	137T	Male	48	Ever-smoker (110)	EML4-ALK	Frozen tissue (surgical specimen)	NGS
	169T	Male	30	Ever-smoker (600)	EML4-ALK	Frozen tissue (surgical specimen)	NGS
	236T	Female	63	Never	EML4-ALK	Frozen tissue (surgical specimen)	NGS
	255T	Female	52	Never	EML4-ALK	Frozen tissue (surgical specimen)	NGS
	L07K098_T	Female	32	Never	EML4-ALK	Frozen tissue (surgical specimen)	NGS
	AD08_351T	Female	38	Ever-smoker (140)	EML4-ALK	Frozen tissue (surgical specimen)	NGS
	AD08_355T	Female	68	Never	EML4-ALK	Frozen tissue (surgical specimen)	NGS
	AD09-218T	Male	42	Never	EML4-ALK	Frozen tissue (surgical specimen)	NGS
	AD09-352T	Male	65	Ever-smoker (700)	EML4-ALK	Frozen tissue (surgical specimen)	NGS
ROS1	103T	Female	40	Never	CD74-ROS1	Frozen tissue (surgical specimen)	NGS
	121T	Male	47	Never	CD74-ROS1	Frozen tissue (surgical specimen)	NGS
	199T	Female	66	Never	CD74-ROS1	Frozen tissue (surgical specimen)	NGS
	L07K147_T	Female	54	Never	EZR-ROS1	Frozen tissue (surgical specimen)	NGS
	AD08_009T	Female	64	Never	CD74-ROS1	Frozen tissue (surgical specimen)	NGS
	AD08_034T	Female	56	Never	EZR-ROS1	Frozen tissue (surgical specimen)	NGS
	AD08_047T	Female	59	Never	CD74-ROS1	Frozen tissue (surgical specimen)	NGS
	AD09-074T	Female	68	Never	CD74-ROS1	Frozen tissue (surgical specimen)	NGS
	AD09-224T	Female	55	Never	CD74-ROS1	Frozen tissue (surgical specimen)	NGS
	AD09-230T	Female	61	Never	CD74-ROS1	Frozen tissue (surgical specimen)	NGS
	AD09-254T	Male	59	Ever-smoker (220)	CD74-ROS1	Frozen tissue (surgical specimen)	NGS
	AD09-466T	Female	60	Ever-smoker (600)	EZR-ROS1	Frozen tissue (surgical specimen)	NGS

Table S1. *Cont.*

Onco-gene	Name	Sex	Age	Smoking (Brinkman index)	Fusion	DNA source	Method for breakpoint identification
	BR0020	Male	57	Never	KIF5B-RET	Frozen tissue (surgical specimen)	Genomic PCR
	L07K201T	Male	64	Ever-smoker (1,350)	KIF5B-RET	Frozen tissue (surgical specimen)	Genomic PCR
	349T	Male	61	Ever-smoker (1,000)	KIF5B-RET	Frozen tissue (surgical specimen)	Genomic PCR
	AD08-341T	Female	39	Never	KIF5B-RET	Frozen tissue (surgical specimen)	Genomic PCR
	RET-024	Male	67	Ever-smoker (320)	CCDC6-RET	MFPE tissue (surgical specimen)	NGS
	RET-030	Female	54	Never	CCDC6-RET	MFPE tissue (surgical specimen)	NGS
	AD12-106T	Male	78	Ever-smoker (550)	KIF5B-RET	Frozen tissue (surgical specimen)	Genomic PCR
RET	BR0030	Male	57	Never	KIF5B-RET	Frozen tissue (surgical specimen)	Genomic PCR
	442T	Male	57	Never	KIF5B-RET	Frozen tissue (surgical specimen)	Genomic PCR
	AD08-144T	Female	39	Never	KIF5B-RET	Frozen tissue (surgical specimen)	Genomic PCR
	BR1001	Female	65	Never	KIF5B-RET	Frozen tissue (surgical specimen)	Genomic PCR
	BR1002	Female	64	Never	KIF5B-RET	Frozen tissue (surgical specimen)	Genomic PCR
	BR1003	Male	28	Never	KIF5B-RET	Frozen tissue (surgical specimen)	Genomic PCR
	AD09-369T	Female	74	Never	KIF5B-RET	Frozen tissue (surgical specimen)	Genomic PCR
	AD12-001T	Female	49	Ever-smoker (580)	KIF5B-RET	Frozen tissue (pleural effusion)	Genomic PCR

Abbreviations: ADC, adenocarcinoma; MFPE, methanol fixed paraffin embedded; FFPE, formalin fixed paraffin embedded; NGS, next generation sequencing. [a] Fused exon numbers of the EML4 (E), ALK (A), CD74 (C) , EZR (E), ROS1 (R) KIF5B (K) , CCDC6 (C) and RET (R) genes.

Table S2. Location of breakpoints for fusions in lung adenocarcinoma on Human Dec. 2013 (GRCh38/hg38) Assembly.

Onco-gene	Name	Fusion	locus in EML4/ CD74/EZR/ KIF5B/CCDC6	Location of breakpoints	Flanking sequence [a] (Upstream of breakpoint)	Locus in ALK/ ROS1/RET	Location of breakpoints	Flanking sequence [a] (Downstream of breakpoint)
ALK	L07K165_T	EML4-ALK	intron 5	chr2:42,279,667-42,279,668	tagagacggg	intron 19	chr2:29,224,457-29,224,458	ggatttttca
		ALK-EML4	intron 5	chr2:42,279,684-42,279,685	acgatggtct *	intron 19	chr2:29,224,461-29,224,462	ggtagagctc **
	AD09-357T	EML4-ALK	intron 5	chr2:42,268,744-42,268,745	gcctacattt	intron 19	chr2:29,225,368-29,225,369	tcgttatgct
		ALK-EML4	intron 5	chr2:42,268,748-42,268,749	gagatggaaa *	intron 19	chr2:29,225,367-29,225,368	ggtgtcactt **
	L07K154_T	EML4-ALK	intron 5	chr2:42,271,264-42,271,265	catctgggct	intron 19	chr2:29,224,614-29,224,615	actcatttgc
		ALK-EML4	intron 5	chr2:42,271,264-42,271,265	acttgtccaa *	intron 19	chr2:29,224,612-29,224,613	agcgggtgac **
	AD09-055T	EML4-ALK	intron 5	chr2:42,276,709-42,276,710	ggtagtatta	intron 19	chr2:29,224,571-29,224,572	cacagaatct
		ALK-EML4	intron 5	chr2:42,276,709-42,276,710	tcactttatg *	intron 19	chr2:29,224,517-29,224,518	ggagtttgcc **
	43T	EML4-ALK	intron 5	chr2:42,277,317-42,277,318	acaaggaaat	intron 19	chr2:29,224,998-29,224,999	ttcagcctgt
	137T	EML4-ALK	intron 5	chr2:42,271,219-42,271,220	tactgtccct	exon 19	chr2:29,225,510-29,225,511	CTCTGCCCTC
	169T	EML4-ALK	intron 12	chr2:42,295,576-42,295,577	ctttcagtcc	intron 19	chr2:29,224,389-29,224,390	agtggttagg
	236T	EML4-ALK	Exon 13	chr2:42,301,389-42,301,390	AAGAGAAATA	intron 19	chr2:29,224,929-29,224,930	tactgctggc
	255T	EML4-ALK	Exon 13	chr2:42,301,325-42,301,326	TGTTATTAAC	intron 19	chr2:29,224,177-29,224,178	aacacaactg
	L07K098_T	EML4-ALK	intron 5	chr2:42,278,956-42,278,957	aatgtattga	intron 19	chr2:29,225,450-29,225,451	agagccccag
	AD08_351T	EML4-ALK	intron 12	chr2:42,299,727-42,299,728	tcactcttgt	intron 19	chr2:29,225,132-29,225,133	ccacatgctt
	AD08_355T	EML4-ALK	intron 12	chr2:42,296,973-42,296,974	ttttcaaatc	intron 19	chr2:29,224,670-29,224,671	aatctgatca
	AD09-218T	EML4-ALK	intron 17	chr2:42,316,483-42,316,484	tctagtgcat	intron 19	chr2:29,223,692-29,223,693	acaccttctt
	AD09-352T	EML4-ALK	intron 12	chr2:42,298,354-42,298,355	tgtgattaag	intron 19	chr2:29,224,312-29,224,313	cctggtcctc
ROS1	103T	CD74-ROS1	intron 6	chr5:150,404,135-150,404,136	ctaggattca	intron 33	chr6:117,324,616-117,324,617	agcaaaaaca
		ROS1-CD74	intron 6	chr5:150,404,099-150,404,100	tgcctggggc *	intron 33	chr6:117,324,575-117,324,576	gcataaacac **
	121T	CD74-ROS1	intron 6	chr5:150,404,542-150,404,543	ctcacttctt	intron 33	chr6:117,326,072-117,326,073	taaactattc
	199T	CD74-ROS1	intron 6	chr5:150,403,318-150,403,319	atgcctgtcg	intron 33	chr6:117,324,718-117,324,719	tggtcctaaa
	L07K147_T	EZR-ROS1	exon 11	chr6:158,769,917-158,769,918	CAGAGGGCCC	intron 31	chr6:117,331,407-117,331,408	cccacgtgtt
	AD08_009T	CD74-ROS1	intron 7	chr5:150,402,876-150,402,877	cctttcatct	intron 33	chr6:117,324,853-117,324,854	tctacacaac

Table S2. *Cont.*

Onco-gene	Name	Fusion	locus in EML4/ CD74/EZR/ KIF5B/CCDC6	Location of breakpoints	Flanking sequence [a] (Upstream of breakpoint)	Locus in ALK/ ROS1/RET	Location of breakpoints	Flanking sequence [a] (Downstream of breakpoint)
ROS1	AD08_034T	EZR-ROS1	intron 9	chr6:158,770,642-158,770,643	agcgcagact	intron 33	chr6:117,326,167-117,326,168	tatcagtaca
	AD08_047T	CD74-ROS1	intron 6	chr5:150,403,628-150,403,629	gatccgccca	intron 31	chr6:117,329,480-117,329,481	catatcaacc
	AD09-074T	CD74-ROS1	intron 6	chr5:150,404,244-150,404,245	ctcatggaca	exon 33	chr6:117,326,228-117,326,229	TGGAGgtatg
	AD09-224T	CD74-ROS1	intron 6	chr5:150,404,550-150,404,551	ctcagctctc	intron 33	chr6:117,324,755-117,324,756	aatgccaact
	AD09-230T	CD74-ROS1	intron 6	chr5:150,404,049-150,404,050	tacatgctat	intron 32	chr6:117,326,453-117,326,454	attgtcatgt
	AD09-254T	CD74-ROS1	intron 6	chr5:150,403,483-150,403,484	gatctcagac	intron 33	chr6:117,324,926-117,324,927	acaaaatcag
	AD09-466T	EZR-ROS1	intron 9	chr6:158,770,624-158,770,665	aaatgataaa	intron 33	chr6:117,325,585-117,325,586	aatttagttg
RET	BR0020	KIF5B-RET	intron 15	chr10:32,026,223-32,026,224	cttgatctcc	intron 11	chr10:43,116,265-43,116,266	cctgggctgg
	L07K201T	KIF5B-RET	intron 15	chr10:32,025,255-32,025,256	aatggtcttc	intron 11	chr10:43,116,578-43,116,579	catagGAGGA
	349T	KIF5B-RET	intron 15	chr10:32,024,847-32,024,848	tggtccgcct	intron 11	chr10:43,115,230-43,115,231	ggtgtgagtg
	AD08-341T	KIF5B-RET	intron 15	chr10:32,024,672-32,024,673	agcaattctt	intron 11	chr10:43,115,128-43,115,129	cctctcctgg
	RET-024	CCDC6-RET	intron 1	chr10:59,856,493-59,856,494	gactgagatg	intron11	chr10:43,115,739-43,115,740	tatttgagag
	RET-030	CCDC6-RET	intron 1	chr10:59,902,926-59,902,927	tagggcatct	intron11	chr10:43,116,231-43,116,232	agttgtcctg
	AD12-106T	KIF5B-RET	intron 15	chr10:32,026,467-32,026,468	tctgttagag	intron 11	chr10:43,115,747-43,115,748	aggatcaggt
	BR0030	KIF5B-RET	intron 16	chr10:32,022,515-32,022,516	tcacaaggag	intron 11	chr10:43,116,133-43,116,134	gtgcgtgagg
	442T	KIF5B-RET	exon 23	chr10:32,018,127-32,018,128	ATGACACCGG	intron 11	chr10:43,114,743-43,114,744	tccctgcggg
	AD08-144T	KIF5B-RET	intron 15	chr10:32,023,512-32,023,513	acagttttat	intron 11	chr10:43,116,139-43,116,140	gagggccagt
	BR1001	KIF5B-RET	intron 15	chr10:32,027,877-32,027,878	ctcaagcctg	intron 11	chr10:43,115,842-43,115,843	gaagccatgg
	BR1002	KIF5B-RET	intron 15	chr10:32,026,093-32,026,094	tgattgttgt	intron 11	chr10:43,114,798-43,114,799	ggccagctgg
	BR1003	KIF5B-RET	intron 23	chr10:32,017,899-32,017,900	ataagtaact	intron 11	chr10:43,116,571-43,116,572	tttccaacat
	AD09-369T	KIF5B-RET	intron 15	chr10:32,027,460-32,027,461	caatgaactc	intron 11	chr10:43,115,221-43,115,222	ctcagagcca
	AD12-001T	KIF5B-RET	intron 15	chr10:32,025,925-32,025,926	tttatagtat	intron 12	chr10:43,117,069-43,117,070	gagaccacca

Nucleotides in exons are in capitals. *: Downstream of breakpoint. **: Upstream of breakpoint.

© 2015 by the authors; licensee MDPI, Basel, Switzerland. This article is an open access article distributed under the terms and conditions of the Creative Commons Attribution license (http://creativecommons.org/licenses/by/4.0/).
